# Effectiveness of teaching psychopathology through the analysis of movie characters: a randomized controlled trial in Shandong Province, China

**DOI:** 10.1038/s41598-023-37949-6

**Published:** 2023-07-04

**Authors:** Yun Song, Yingjie Ma, Yi Huang, Yang Wang, Peiru Xu, Guangchuan Huang, Xu Chen

**Affiliations:** 1 Department of Neurology, The First Affiliated Hospital of Shandong First Medical University and Shandong Provincial Qianfoshan Hospital, Jinan, 250014 China; 2 Department of Psychiatry, Shandong Mental Health Center, Shandong University, 12 Ward of Psychiatry, Inpatient Building, 49 Wenhua East Road, Jinan, 250014 China; 3 School of Humanities and Social Sciences, Binzhou Medical University, Yantai, 264003 China; 4 Department of Psychiatry, Cheeloo College of Medicine, Shandong University, Jinan, 250014 China; 5 College of Mental Health, Jining Medical University, Jining, 272000 China

**Keywords:** Psychology, Health occupations

## Abstract

We studied the effectiveness of movie character analysis for teaching psychotic symptomatology to medical undergraduates. We randomly selected two of six medical schools in Shandong Province, China, then randomly assigned eight undergraduate classes at those schools to intervention or control groups. The intervention group (n = 162) participated in seminars in which psychotic symptoms were explored through analysis of movie characters. The control group (n = 165) participated in conventional seminars. The participants in both groups were surveyed with a custom-designed questionnaire, and their knowledge was assessed using a written exam. Compared to the control group, the intervention group showed greater interest in the topic (t = 5.63, p < 0.001), better understanding of psychotic symptoms (t = 2.37, p = 0.02), and greater acceptance (t = 9.80, p < 0.001). In addition, the intervention group showed significantly greater knowledge on the written exam (t = 5.78, p < 0.001). Analyzing movie characters can improve the teaching of psychotic symptomatology and should be further explored and promoted.

## Introduction

Films can leave deep impressions on viewers due to the detailed and vivid representation of characters^1^. As a result, films have become popular teaching tools among medical educators^2,3^, giving rise to the field of “cinemeducation”^4^. Cinemeducation has been found to be more effective than conventional seminars for teaching many aspects of medical humanities, especially medical ethics and doctor-patient relations^5^. Cinemeducation can help students see how theory plays out in practice, as well as cultivate skills such as empathy and self-reflection^5^.

Cinemeducation has also proven useful for teaching aspects of psychiatry^6,7^. Films can be used to depict concrete situations that force students to engage with certain problems and propose solutions, improving their ability to diagnose and treat diseases^8,9^. Cinemeducation allows the exploration of clinical problems from multiple angles within a safe, ethical environment^10,11^, which may be impossible to achieve otherwise during conventional clinical training^3,12^. Indeed, this teaching format allows pausing and repetition to promote deeper consideration of certain topics^3,12,13^.

Some studies have explored the potential of cinemeducation for teaching psychopathology to medical students, including in China. In those studies, cinemeducation projects were used to help psychiatry residents and medical undergraduate students understand abnormal psychological symptoms^14–18^, reduce prejudice or stigma against mental illness^19^, and improve medical professionalism^7,12^. Cinemeducation supported those goals effectively in that previous work, suggesting the power of combining films or movie clips with lectures and patient interviews. That literature, however, suffers from the disadvantages of small samples^7,14,15^ or lack of objective evaluation criteria^12,16^, highlighting the need for further work in this area.

Here we propose the analysis of movie characters as an approach to teaching psychotic symptomatology in medical school. In this approach, movie clips serve as the basis for explaining symptoms. The analysis of movie characters during scenes shown in class may allow students to understand a patient’s subjective experience and understand psychiatric symptoms more deeply than can be achieved through conventional seminars. To evaluate the efficacy of this approach objectively and avoid the disadvantages of some previous studies, the present work applied a randomized controlled design. In this way, the present study may be the largest, most detailed analysis of movie character analysis to teach psychopathology in China.

## Subjects and methods

### Subjects

Using a random number table, we randomly selected two of the six universities in Shandong Province with regular full-time clinical medicine programs, because the investigators have the greatest experience with medical education in this province. The two medical schools ranked 17th (Cheelo College of Medicine, Shandong University) and 94th (Jining Medical University) among 150 medical schools in China in 2021^20^, suggesting that they can be taken as representative of schools of extremely high and intermediate quality. At each of the two schools, we selected four fourth-year (penultimate year) medical school classes in the second semester of the 2018–2019 academic year. Using random number tables, we allocated the total of 332 enrolled undergraduates to an intervention group (n = 164) and control group (n = 168).

### Ethics approval

Before the study began, all students provided written informed consent to participate. The study protocol was approved by the Ethics Committee of Shandong Mental Health Center (2017R035). All methods were carried out in accordance with the CONSORT 2010 guidelines.

### Teaching methods

The intervention group was shown clips from three English-language movies subtitled in Chinese during the course, which served as the basis for explaining and analyzing mental symptoms. The showing of the clips and their discussion lasted two teaching hours (100 min). The films were selected because psychopathology was a central theme and either the film itself (A Beautiful Mind) or main actor(s) had received an Academy Award Oscar (Leonardo DiCaprio for The Revenant and Natalie Portman for Black Swan).We did not consider movies that the Chinese National Film Board had ruled could be viewed only by those 18 years and older. The movies were carefully reviewed by the investigators before the research began, and appropriate clips were selected that did not include excessive violence or eroticism. Psychopathological symptoms that were not illustrated in the clips, such as those related to attention, memory, intelligence, affection, volition and behavior, were taught in conventional seminars lasting another two teaching hours.

The following three characters from three movies were analyzed: Teddy Daniels from *Shutter Island,* directed by Martin Scorsese; John Forbes Nash, Jr. from *A Beautiful Mind,* directed by Ron Howard; and Nina Sayers from *Black Swan,* directed by Darren Aronofsky. The clips shown from these movies lasted a total of 15 min.

Teddy in *Shutter Island* experiences vivid, rich visual and auditory hallucinations. To explain the difference between hallucinations and dreams, we showed a clip from the movie (27 min 28 s–30 min 15 s) in which Teddy speaks with his deceased wife. Teddy clearly sees his deceased wife standing before him and burning into ashes. The lecturers used this clip to illustrate the phenomenological quality of auditory or visual hallucinations as a typical psychotic symptom if it had been experienced in the waking state in which perceptual experience does not correspond with reality. With the aid of this video clip, we explained to the students the characteristics of visual and auditory hallucinations, the differences between perceptual and sensory experiences, and the distinction between dreams and hallucinations.

John in *A Beautiful Mind* experiences visual hallucinations, auditory hallucinations as well as delusions of being persecuted, followed and monitored. We showed a film clip in which John speaks with a mysterious federal agent (32 min 33 s–34 min 45 s), who is actually a visual hallucination that does not exist. We also showed clips in which John waves his hands to pointing the handwriting in windows and speaks to the non-existent man in library (11 min 25 s–12 min 54 s), and in which he cuts his arm in his effort to find the monitoring device that he believes has been implanted there (1 h 16 min 00 s–1 h 18 min 18 s). We used these clips to illustrate the characteristics of different types of delusional thoughts and behaviors from the patient’s perspective.

The ballerina Nina in *Black Swan* experiences delusions and resulting emotional and behavioral disorders. We showed film clips depicting Nina's anxiety, fear, and irritability due to her delusions (1 h 15 min 00 s–1 h 22 min 50 s). Nina thought Lily and other ballerinas in this opera troupe always did harm to her for taking place of herself. When Nina was informed of that Lily was her understudy, she thoroughly freaked out. Those caused Nina being on tenterhooks, fear and irritableness. We used Nina's various emotional expressions to explain emotional disorders, and we used her psychosis-induced self-injury behaviors to illustrate volitional disorders and the resulting hyperbulia and volitional ambivalence.

The control group participated in conventional seminars on psychiatric symptomatology, lasting four teaching hours (200 min), in which instructors explained the same symptoms of psychopathology as emphasized in the intervention group, but involving the reading of relevant excerpts from the textbook *Psychiatry* (8th edition)^21^ instead of viewing film clips. Thus, the intervention and control groups received the same educational content, with the sole difference that the intervention group drew on movie clips instead of text excerpts to illustrate certain symptoms.

To ensure consistency in teaching methods, two associate professors and one lecturer were trained in how to analyze the film characters before the study began. All instructors in this study had at least five years of experience teaching psychiatry.

### Evaluation methods

We custom-designed a self-report questionnaire to collect data on student demographics, and scholarships awarded, as well as their understanding of, acceptance of, and interest in the two teaching methods.This questionnaire can be downloaded as following (Supplementary Information). Demographic data included gender, age, and place of residence (urban or rural). Data on scholarships attained served as a proxy for academic performance in order to detect differences between the intervention and control groups; only school-level or higher-level scholarships were considered, not subject-specific awards. Interest, understanding and acceptance were assessed subjectively using a 10-point scale ranging from 0 (lowest) to 9 (highest) similar to a visual analogue scale^22^. The respondents should select the fitted scores based on their subjective feelings. The mutual correlations in duplicate measurement on interest, understanding and acceptance are respectively 0.97, 0.98 and 0.96 in preliminary experiment, which have 43 students uninvolved this trial as the sample before the trial. And the teaching topic of preliminary experiment was not on psychiatry. The 30 questions were designed to evaluate knowledge about psychopathology and they were not tailored or even focused on the topics in the film clips. Students completed the questionnaires after the conventional seminars or the intervention. The questionnaires did not bear any individual identifying information to ensure anonymity.

The effectiveness of the teaching methods was also assessed using 30 questions randomly selected from the corresponding medical school’s standard databank of questions for assessing knowledge of psychopathology. The paper-based exam comprised 20 single-choice questions and 10 multiple-choice questions, and each of the 30 questions was worth 1 point. Thus, the total possible score was 30 points. After the students completed the assessment, they were told that their scores would not be included in the overall assessment of their academic performance.

### Assessments

Immediately after completing the conventional or movie character-based seminars, students completed the questionnaire and knowledge exam on-site. During the exam, students were not allowed to consult books or communicate with others. Both assessments were completed within 30 min.

### Statistical testing

Data were entered into an Excel spreadsheet (Microsoft Office), analyzed using SPSS 17.0 (IBM, Chicago, IL, USA), and reported as mean ± standard deviation. Data were tested for normal distribution, then inter-group differences were assessed for significance using chi-squared or *t* tests as appropriate. Differences were considered significant at α = 0.05.

## Results

### Summarizing of assessments

**Figure 1:**
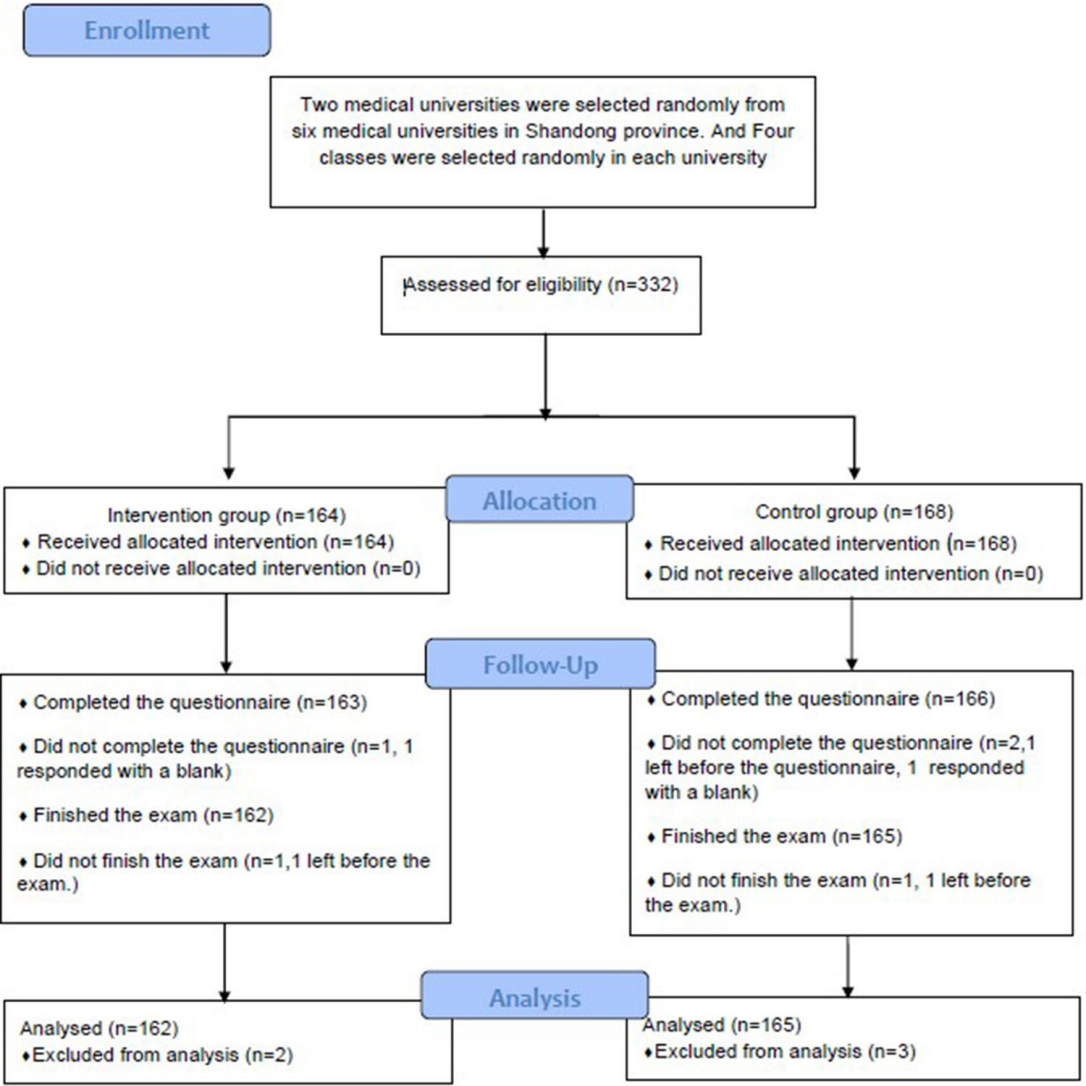
Flowchart of the study.

Of the 330 questionnaires distributed, 329 were returned (99.7% return rate), among which 327 were valid (99.1% completion rate). Two were invalid because they were completely blank. Of the 329 exams issued, 327 were returned (99.4% return rate) and all were valid (100% completion rate). In the control group, one student left before completing the questionnaire. One student in each group withdrew from the study for personal reasons before completing the knowledge exam. The final analysis included 327 undergraduates with complete data, 162 in the intervention group and 165 in the control group (Fig. 1).

### Comparison of intervention and control groups

The two groups did not differ significantly in gender distribution (χ^2^ = 0.94, p = 0.96) or age (t = 1.56, p = 0.12; Table 1). They also did not differ significantly in proportions of students from urban or rural locations, or in the proportion with scholarships (χ^2^ = 0.14, p = 0.97).

**Table 1:** Comparison of basic characteristics between the intervention and control groups.

Characteristic	Control group (n = 165)	Intervention group (n = 162)	*χ*^*2*^ or *t*
Gender (male/female)	79/86	77/85	0.94*
Age, year	21.47 ± 0.97	21.65 ± 1.14	1.56*
Birthplace (urban/rural)	98/67	87/75	1.25*
Scholarship recipients, n (%)	53 (32.12)	50 (30.86)	0.14*

*p > 0.05.

### Effectiveness of teaching based on movie character analysis

**Figure 2:**
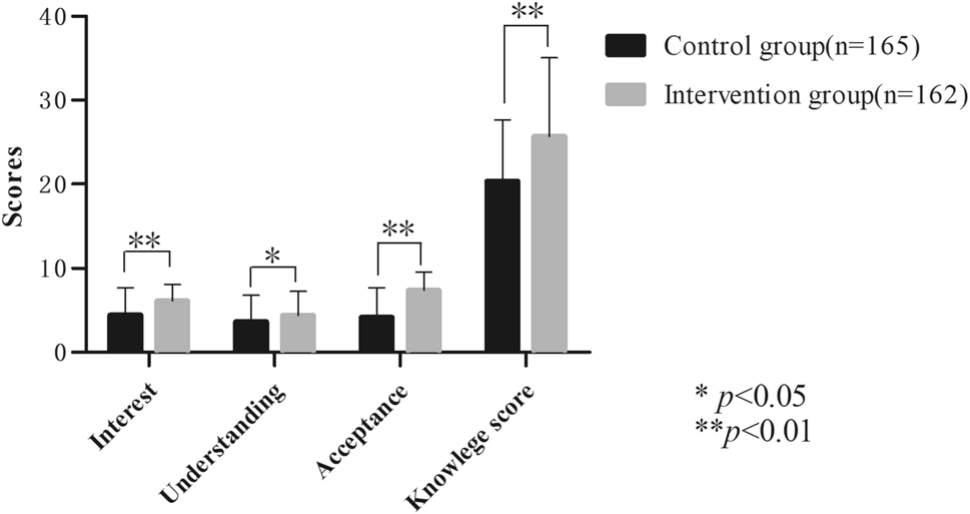
Comparison of questionnaire and exam scores between the intervention and control groups.

Students in the intervention group showed significantly greater interest (t = 5.63, p < 0.001), understanding (t = 2.37, p = 0.02) and acceptance (t = 9.80, p < 0.001) than those in the control group (Fig. 2). Students in the intervention group also scored significantly higher on the knowledge exam (t = 5.78, p < 0.001).

## Discussion and conclusion

With the rapid development of Chinese society, competitiveness, pressures and conflicts between what people desire and external reality have increased the prevalence of mental problems and disorders. This and the pandemic of coronavirus disease 2019 have exacerbated the preexisting shortage of mental healthcare professionals. The Chinese government issued the National Mental Health Work Plan (2015–2020)^23^, which calls for doubling the number of assistant psychiatrists in psychiatric departments, but the success of the Plan depends on strengthening the ability of general healthcare personnel to identify mental illnesses. In this regard, psychiatric training is a particularly important part of medical education in China. It should lie on a par with internal medicine and surgery as one of the foundations of clinical training^24^.

Psychopathology is a bridge linking basic medicine and clinical psychiatry, just as pathology and pathophysiology are “bridge” subjects linking basic and clinical medicine. Teaching psychopathology to medical students is challenging because few students have been exposed to mental illness. This is particularly true in China, where medical students are undergraduates, in contrast to the older postgraduates in many Western countries^25^. As a result, they may not easily understand psychiatric symptoms from the concise and general descriptions in textbooks, so the symptoms remain abstract, subjective and potentially confusing^26^.

To address this problem, we are aware of at least one textbook, *Psychiatry* (8th edition), edited by Wei Hao^21^, provides short video clips that illustrate symptoms. While such efforts can improve the teaching of psychopathology, video clips lasting one minute cannot adequately illustrate the symptoms in depth, their causes and effects, or progression of the disease. A deep understanding of psychiatric symptoms requires knowledge of the individual’s medical history, since patients who present the same symptoms but have different histories may require different diagnoses and treatments. Focusing only on symptoms and ignoring the individual as a whole defeat the purpose of patient-centered, individualized medicine.

Precisely because of these limitations, some psychopathology courses have allowed students to attend mental examinations, during which some patients with typical psychiatric symptoms are interviewed. While this can deepen students’ understanding of symptoms, it may induce stigma in patients and lead to uncontrollable or unpredictable reactions, including agitation, impulsive behavior, silence, rejection, or even attempts to escape. This practice may be ethically problematic^27^, especially given the current tense relationship between doctors and patients in China^28^.

An alternative for teaching psychopathology is using movie clips, which are easily accepted and understood by students^29^ and which can vividly portray the distinct symptoms of mental illness much better than text descriptions^4^. Movie character analysis, which combines the sensorial images of movie clips with written descriptions in textbooks, can help students create emotional and intellectual connections to the subject matter. In other words, students can acquire, to a certain extent, subjective perception and emotional experience of psychiatric symptoms by vicariously experiencing them through the movie characters^30^. In this way, movie character analysis may increase students' acceptance of, and interest in, psychopathology, ultimately helping the students focus on their studies and remember key learning points. Movie character analysis effectively alternates between abstract psychiatric concepts and visualization, facilitating memorization by undergraduates without a foundation in psychology.

At the same time, using analysis of movie characters to teach psychopathology poses some challenges. For example, appropriate characters and film clips should be chosen carefully and dialectically, and students should remain focused on the symptomatology and not lose sight of learning objectives by getting lost in the plot. Since passive watching is not the same as active learning, teachers should carefully balance the visual and emotional effects of the movie clips against a “dry” theoretical approach. Furthermore, clips are visual media in which visual symptoms such as hallucinations may be clearer and more memorable to students than symptoms involving other senses, such as auditory hallucinations. For example, the clips in *A Beautiful Mind* show visual and auditory hallucinations at the same time, but the visual ones may seem much more vivid to students, who may therefore neglect audio symptoms.

Our work confirms and extends several previous studies^19,23,31,32^ demonstrating that movie character analysis can be superior to traditional teaching methods for creating understanding and acceptance of, as well as interest in, psychopathology among medical students.

Our study is limited by the fact that we used an unvalidated self-report tool to assess interest, acceptance and understanding of the field. It is also limited by our relatively small sample, and the fact that we did not consider potential gender differences. Therefore, our findings should be verified and extended in larger studies that involve a broader range of objective assessments, preferably which have already been validated for accuracy and reliability, and that compare genders.

## Supplementary Information

Supplementary Information.

## Data Availability

The data that support the findings of this study are available on request from the corresponding author. The data are not publicly available due to privacy or ethical restrictions.
