# Supplementary material for: Effectiveness of teaching psychopathology through the analysis of movie characters: a randomized controlled trial in Shandong Province, China
Source: Sci Rep. 2023 Jul 4;13:10800. doi: 10.1038/s41598-023-37949-6 (PMC10319716; doi:10.1038/s41598-023-37949-6)
Supplement: Supplementary file 1 — Supplementary Information. [file 41598_2023_37949_MOESM1_ESM.pdf]

***This survey is anonymous. Please select or fill in the following information according to your actual situation. Thanks for your cooperations.***

Gender: female / male Age: ______years

Place of your family residence: urban / rural

Have you attained the school-level or higher-level scholarships during your college time? Yes / no

***Based on your personal feelings in the classes of psychopathology, please choose the scores that suits you best in the below form (Increasing from left to right).***

| | | | | | | | | | | |
|---|---|---|---|---|---|---|---|---|---|---|
| **Interest** | 0 | 1 | 2 | 3 | 4 | 5 | 6 | 7 | 8 | 9 |
| **Understanding** | 0 | 1 | 2 | 3 | 4 | 5 | 6 | 7 | 8 | 9 |
| **Acceptance** | 0 | 1 | 2 | 3 | 4 | 5 | 6 | 7 | 8 | 9 |
| | ***none*** | | | | | | | | | ***fully*** |
